# Transcranial alternating current stimulation as treatment for delirium: A multicenter randomized double-blind sham-controlled pilot study on safety and feasibility

**DOI:** 10.1016/j.neurot.2026.e01037

**Published:** 2026-08-04

**Authors:** Julia van der A, D. Yorben Lodema, Edwin van Dellen, Indira Tendolkar, Marielle H. Emmelot-Vonk, Dennis J.L.G. Schutter, Willem de Haan, Mark van den Boogaard, Thomas H. Ottens, Arjen J.C. Slooter

**Affiliations:** a Department of Intensive Care Medicine and University Medical Center Utrecht Brain Center, University Medical Center Utrecht, Utrecht University, Utrecht, the Netherlands; b Department of Psychiatry and University Medical Center Utrecht Brain Center, University Medical Center Utrecht, Utrecht University, Utrecht, the Netherlands; c Department of Psychiatry, University Medical Center Groningen and Brain Center for Cognitive Neuroscience, University of Groningen, Groningen, the Netherlands; d Department of Neurology, UZ Brussel and Vrije Universiteit Brussel, Brussels, Belgium; e Donders Institute for Brain, Cognition and Behavior, Department of Psychiatry, Radboud University, Nijmegen, the Netherlands; f Department of Geriatrics, University Medical Center Utrecht, Utrecht University, Utrecht, the Netherlands; g Experimental Psychology, Helmholtz Institute, Utrecht University, Utrecht, the Netherlands; h Alzheimer Center and Department of Neurology, Amsterdam Neuroscience, VU University Medical Center, Amsterdam UMC, Amsterdam, the Netherlands; i Department of Intensive Care Medicine, Radboud University Medical Center, Nijmegen, the Netherlands; j Intensive Care Unit, HagaZiekenhuis, The Hague, the Netherlands

**Keywords:** Delirium, Transcranial alternating current stimulation, Acute encephalopathy, Non-invasive brain stimulation, Randomized controlled trial

## Abstract

Delirium is an acute neuropsychiatric syndrome with currently no effective treatment. The acute encephalopathy underlying delirium is characterized by diffuse oscillatory slowing on electroencephalography (EEG). Based on its capacity to modulate oscillatory activity, 10 Hz transcranial alternating current stimulation (tACS) is a potential hypothesis-driven intervention for delirium. Before investigating potential neurophysiological or clinical effects, it is necessary to establish whether tACS is feasible and safe in delirious patients. This pilot study investigated the feasibility and safety of tACS in 30 hospitalized patients aged ≥50 years with delirium. Participants were randomized double-blind to receive either daily 10 Hz frontal-occipital tACS or sham stimulation for up to 14 days. The first-session completion rate was 94% (active) and 92% (sham). Participants had a median of 3 eligible treatment days (interquartile range [IQR]:2–4). The active group completed a median of 2 tACS sessions (IQR:2–3) and the sham group a median of 3 (IQR:2–4), corresponding to treatment adherence rates of 69% (95% confidence interval [CI]:57–79) and 73% (95% CI:59–83), respectively. The intervention was well-tolerated with no stimulation-related serious adverse events (AEs). AE frequency did not differ between active (35%) and sham (31%) groups. Side effects were mild, transient cutaneous sensations. In conclusion, short-course exposure to 10 Hz tACS can be safely initiated in hospitalized older adults with delirium, with a high first-session completion rate while delivery of repeated daily sessions remained challenging. These findings support further investigation of tACS in delirium, including studies specifically designed to evaluate neurophysiological and clinical effects. **Trial Registration:** NCT06285721, registered on 2024-02-19

## Introduction

Delirium is a prevalent neuropsychiatric syndrome characterized by an acute disturbance in attention and awareness with additional cognitive dysfunction due to a somatic condition [1]. It affects up to 31% of critically ill patients and 25–50% of all hospitalized older adults [2,3]. Delirium is a risk factor for treatment-associated complications such as auto-extubations, prolonged hospitalization [4] and long-term cognitive impairment including dementia [[5], [6], [7]], contributing to increased healthcare costs associated with delirium [8].

From a pathophysiological perspective, delirium is considered one of the clinical manifestations of acute encephalopathy, which presents with a characteristic electrophysiological pattern [9]. Electroencephalography (EEG) studies show that acute encephalopathy presenting as delirium is associated with a shift toward slower oscillatory frequencies, reflected by reduced peak frequency, increased power in the delta (0.5–4 Hz) and theta (4–8 Hz) bands, alongside decreased power and connectivity in the alpha (8–13 Hz) and beta (13–30 Hz) bands [[10], [11], [12], [13]]. Additionally, the shift to slow wave oscillatory activity correlates with delirium severity [14,15]. Electrophysiological findings in delirium are corroborated by neuroimaging evidence with studies using functional magnetic resonance imaging (fMRI) that have shown impaired integration and efficiency of the default mode network (DMN) in patients with postoperative delirium [16,17] and gray matter volume loss [18]. Crucially, these network alterations are not merely transient. Decreases in global functional connectivity can persist for months following resolution of delirium and are associated with long-term cognitive impairment, which links the acute network dysfunction to long-term negative outcomes [19].

Transcranial alternating current stimulation (tACS), a form of non-invasive brain stimulation that is easy to apply, has emerged as a promising tool for modulating brain activity, first studied in healthy individuals but later also in aging and several neurological and (neuro)psychiatric disorders such as depression and mild cognitive impairment [[20], [21], [22], [23]]. By delivering weak electrical currents, tACS can phase-lock neuronal firing, effectively entraining endogenous rhythms [24,25]. In healthy cohorts, stimulation in the alpha frequency has been shown to upregulate alpha power [26] and strengthen long-range functional connectivity, particularly within the DMN [27]. Furthermore, studies have indicated the potential for tACS to improve cognitive functioning in several cognitive domains, such as attention and working memory [28], domains that are also affected in delirium [29].

Although these findings provide a rationale for investigating whether 10 Hz tACS might be able to modulate brain activity underlying delirium, it remains unknown whether application of tACS in patients with delirium is feasible and safe. Feasibility could be negatively impacted by factors such as agitation or confusion due to retinal phosphenes or simply the procedure of setting up and applying tACS in general. Therefore, the primary objective of this pilot study was to investigate feasibility and safety of administering tACS to patients with delirium. We hypothesized that the application of tACS is feasible in this population, defined as the successful completion of the first scheduled stimulation session by the majority of participants. Furthermore, we hypothesized that the intervention is safe and well-tolerated, anticipating no significant differences in the incidence of serious adverse events ((S)AE), or tACS-specific side effects between the active and sham groups.

## Materials and Methods

### Study setting

This study reports the findings from the pilot phase of the DELTES trial, a multicenter, randomized, double-blind, sham-controlled clinical trial [30]. The trial was conducted across three medical centers in the Netherlands: University Medical Center (UMC) Utrecht, Radboudumc, and HagaZiekenhuis. The protocol was approved by the Medical Ethics Committee of the UMC Utrecht (23–198), registered at ClinicalTrials.gov (NCT06285721, February 19, 2024) and conducted in compliance with the Declaration of Helsinki. Written informed consent was obtained from the patients' legal representatives prior to inclusion. After regaining mental capacity, patients were asked to provide informed consent for ongoing participation and use of previously collected data.

### Participants

We enrolled hospitalized patients aged 50 years or older with a diagnosis of delirium according to the Diagnostic and Statistical Manual of Mental Disorders, Fifth Edition, Text Revision (DSM-5-TR) criteria, as described in detail below. Eligible patients had delirium, lasting at least two days despite adequate treatment of underlying causes, with a Richmond Agitation and Sedation Scale (RASS) score between −2 and +2. The delirium diagnosis was confirmed after study inclusion using formal screening tools and expert consultation (see section 2.4). Exclusion criteria, detailed in the full trial protocol [30], included contraindications for tACS (e.g., pacemaker, metallic brain implants, coils or clips or epilepsy), inability to conduct delirium assessments, stroke or substance withdrawal as the primary cause of delirium, and pre-existing dementia.

### Randomization and blinding

Participants were randomized in a 1:1 ratio to receive active 10 Hz tACS or sham tACS using Castor Electronic Data Capture (Amsterdam, The Netherlands) with permuted blocks of 2 and 4, stratified by study center. Both participants and study personnel assessing outcomes were blinded to treatment allocation. An independent team member, not involved in data collection or analysis, provided the study team members with a specific 5-digit device code that initiated the assigned protocol (active or sham) without revealing the allocation. In patients requiring continuous cardiac monitoring, ECG monitors were shielded from study personnel during stimulation to conceal potential electrical artifacts which otherwise would reveal treatment allocation.

### Study procedures

After informed consent, baseline demographic and clinical data were collected, including frailty assessed using the Clinical Frailty Scale [31] which is a judgment-based frailty tool, ranging from 1 (very fit) to 9 (terminally ill). Delirium duration prior to randomization was estimated based on chart review [32]. Relevant comorbidities were extracted from the electronic patient record. Delirium diagnosis was confirmed using the Delirium Interview [33], a semi-structured interview that operationalizes the DSM-5-TR criteria for delirium, across domains such as level of arousal, attention, orientation and memory. Results were reviewed by a delirium expert (psychiatrist, geriatrician, or intensivist) to confirm the diagnosis before randomization. All participants received standard clinical delirium care according to local protocols and at the discretion of the treating physician. Local delirium protocols in all participating centers were based on contemporary national guidelines [34,35]. The study intervention did not influence routine delirium management.

On the day of the first tACS session, delirium status was assessed using the Intensive Care Delirium Screening Checklist (ICDSC) for ICU patients. For non-ICU patients, both the ICDSC and the Delirium Observation Screening Scale (DOSS) were used, since ICDSC shows limited sensitivity outside the ICU [36]. Then, a 15-min resting-state EEG was recorded using a BioSemi 64-channel ActiveTwo system (for details see Ref. [30]). After this, the first tACS session (active or sham, 30 min) was administered. A second 15-min resting-state EEG was recorded immediately afterward. The EEG cap and electrodes remained in place during the stimulation to minimize patient burden. While these EEG recordings were an integral part of the study protocol, their analysis is outside the scope of this pilot report and will be reported separately. Subjective experiences and any physical sensations (e.g., itching, tingling) of the patients were evaluated to study treatment tolerability, if possible.

**Fig. 1:**
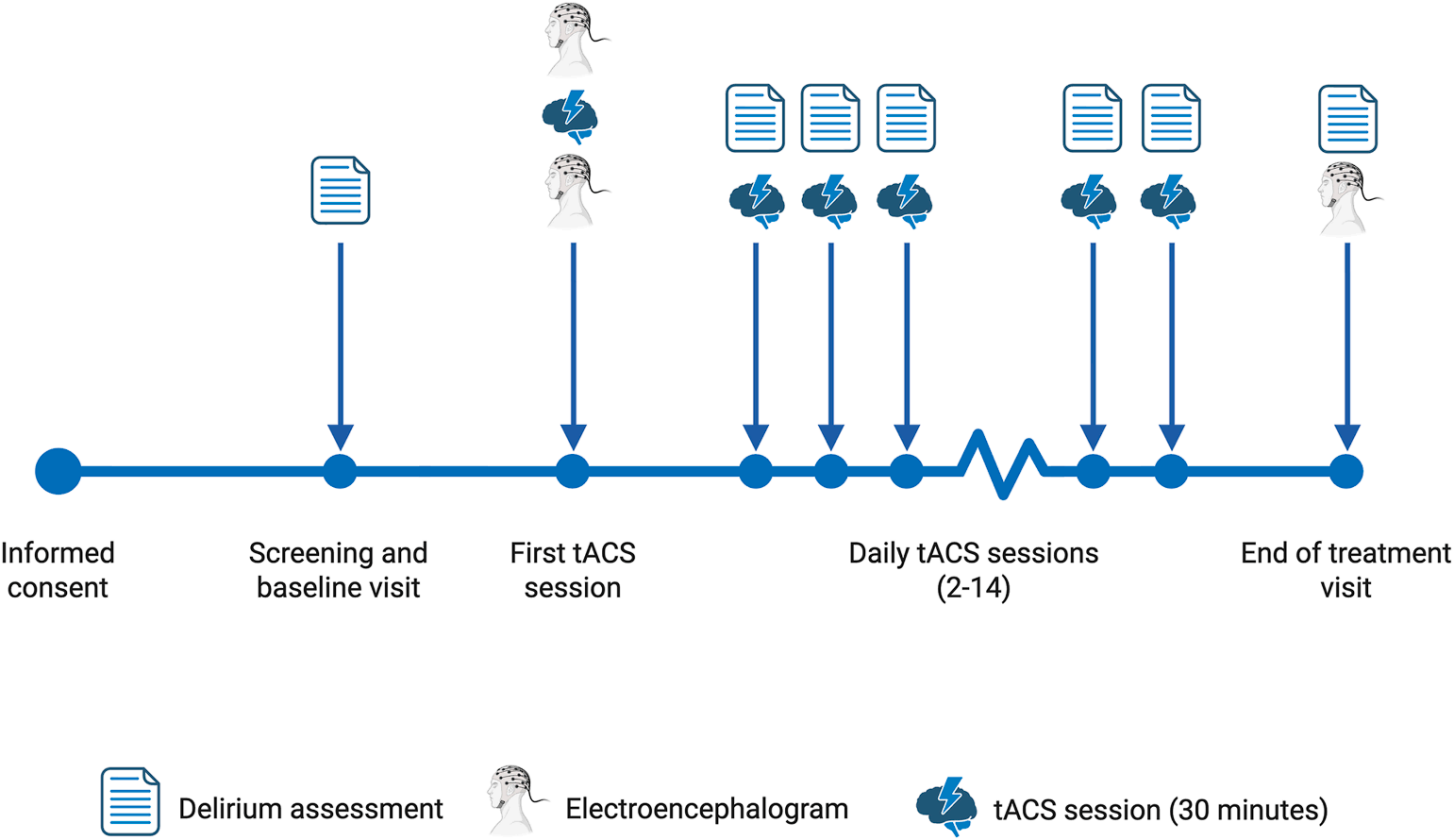
**Study overview**. A timeline shows the consecutive steps and measurements involved in a single inclusion. The number of tACS treatment sessions was variable and based on time to end of delirium or discharge, with a maximum of 14 treatment days. tACS: transcranial alternating current stimulation.

After the first tACS session, participants received daily sham or active tACS for a maximum of 14 days, until hospital discharge or delirium resolution, whichever occurred first. Delirium status was assessed daily to measure delirium duration and to determine treatment continuation. A delirium positive day was defined as an ICDSC score of ≥4 or a DOSS score of ≥3. Delirium resolution was defined as two consecutive days with ICDSC <4 and/or DOSS scores <3 in the ICU and ward, respectively. At the end of the treatment phase, a close-out visit was performed, if possible, including a final 15-min resting-state EEG and a repeated assessment of subjective experiences and sensations. See Fig. 1 for the study overview.

### Intervention

tACS was delivered using a battery-driven electrical current stimulator (Nurostym transcranial electrical stimulation (tES) device, Brainbox, United Kingdom) via two 5 × 5 cm (25 cm^2^ surface area) conductive rubber electrodes enclosed in saline-soaked sponges. Electrodes were positioned according to the international 10-10 EEG system at the AFz (anterior) and Oz (posterior) locations to engage a broad number of anterior-posterior regions (Supplementary Material 1). Participants in the active group received a sinusoidal current at a frequency of 10 Hz (i.e. alpha band) with a peak-to-peak intensity of 2.0 mA. The stimulation session lasted 30 min, including a standard 30-s ramp-up period at the start and a 30-s ramp-down period at the end. To mimic the cutaneous sensations of active stimulation without modulating cortical rhythms, the sham protocol included a 30-s ramp-up to 2.0 mA, followed by a 30-s ramp down to 0 mA, at the beginning and end of the 30 min. No current was delivered for the remainder of the 30-min session in the sham group. Stimulation impedance was maintained at a maximum of 10 kΩ.

### Outcome measures

The primary endpoint was the completion rate of the first session, defined as the percentage of randomized participants who completed the full 30 min stimulation protocol during their first tACS session. To assess feasibility over the full treatment course, we defined three additional metrics. An eligible treatment day was a study day on which the participant remained hospitalized, still met delirium criteria, and research personnel were available (i.e., excluding weekends). A tACS session was considered complete when the full 30-min stimulation protocol was delivered on an eligible treatment day. The treatment adherence rate was the proportion of eligible treatment days on which a complete tACS session was delivered. Recruitment feasibility was monitored by tracking the number of patients screened, eligible, and enrolled per site.

Safety was assessed by comparing the incidence of (S)AEs between groups. Events potentially related to delirium worsening within 1 h of stimulation were specifically monitored, including increased agitation requiring pharmacological intervention or physical restraints, or that resulted in removal of lines or devices. Whether (S)AEs were possibly related to the intervention was determined by a study team member, in consultation with the treating physician where needed, all of whom remained blinded to treatment allocation throughout the trial. Tolerability and side effects were evaluated using a standardized questionnaire administered after the first and last tACS session [30]. The questionnaire assessed both cutaneous sensations (e.g., itching, tingling), other potential side effects (e.g., phosphenes, headache) and subjective discomfort. Sensations were rated on a three-point severity scale (0 = absent, 1 = mild, 2 = severe) and were classified as AEs if they lasted longer than 1 min and were rated as severe. Finally, to evaluate blinding integrity, the researcher present during set-up and stimulation was asked to guess the treatment allocation at the end of the first tACS session. Patient self-reported blinding was attempted but could not be reliably assessed, as a substantial proportion of delirious patients were unable to understand or answer the question.

### Statistical analysis

The first session completion rate was reported as a percentage with 95% confidence intervals (CIs). The treatment adherence rate and number of completed tACS sessions were summarized descriptively per group. Safety data, including (S)AE incidence rates were reported per group. Tolerability and blinding data were summarized descriptively. Feasibility metrics (recruitment rate, adherence measures) were also described descriptively. Practical challenges were summarized qualitatively. Given the pilot nature and small sample size, no formal between-group statistical comparisons were performed. All reports were performed using R (version 4.03).

## Results

### Participant inclusion

**Fig. 2:**
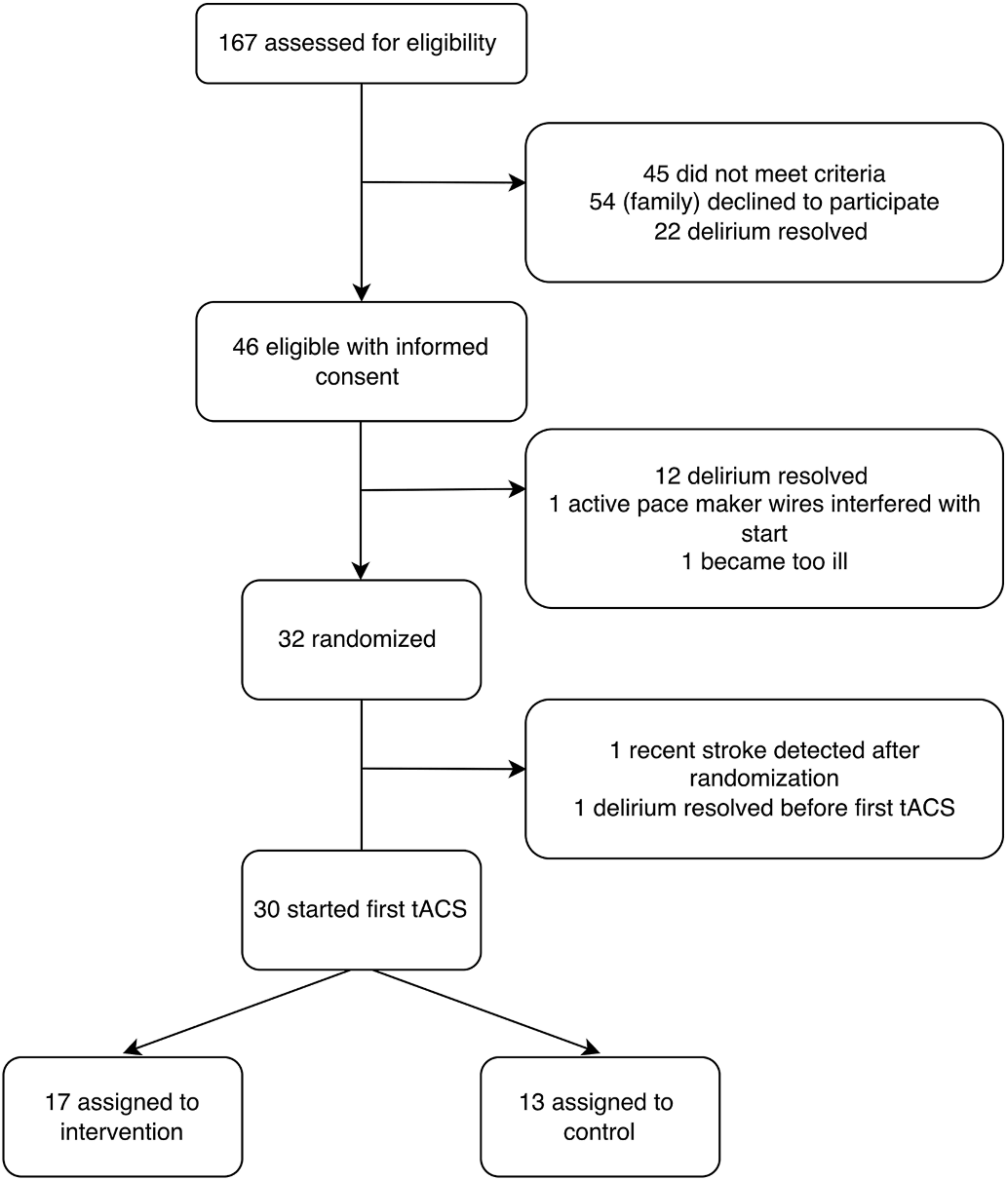
Flow diagram. tACS: transcranial alternating current stimulation.

Between March 2024 and September 2025, 167 patients were assessed for eligibility (Fig. 2).

Of these, 45 (27%) did not meet all inclusion criteria, 54 (32%) declined to participate, and in 22 (13%) delirium had resolved before informed consent could be obtained. For the remaining 46 patients, written informed consent was provided. Prior to randomization, fourteen were excluded: delirium had resolved in twelve, one had active pacemaker wires (a contraindication for tACS), and one became too ill to continue. This left 32 patients who were randomized across three Dutch hospitals: UMC Utrecht (n = 13), HagaZiekenhuis (n = 12), and Radboudumc (n = 7). A further two patients were excluded after randomization: one due to a newly detected stroke, and one because delirium had resolved prior to the start of the first tACS session. In total, 30 patients started treatment with either active (n = 17) or sham tACS (n = 13). This difference in group sizes resulted from the use of permuted-block randomization stratified by study center. Median time from informed consent to first tACS session was 1 day (IQR 0–1, range 0–15). See Fig. 2 for the inclusion flow chart.

All 30 randomized participants received at least one (partial) session of the allocated intervention and were included in the safety and feasibility analyses. Baseline demographic and clinical characteristics were well-balanced between the two groups, as shown in Table 1. The mean age of participants was 74.2 (SD = 9.94) years. The majority of patients were male (83%). The cohort presented with a median clinical frailty scale score of 3 (IQR 3–4). Approximately half of the participants were admitted to the ICU at study initiation (57%), 63% had undergone surgery during current admission, and the majority (73%) were classified as ASA score 3 or 4. The estimated median duration of delirium prior to randomization was 4.5 days [IQR 3–7]. Two-thirds of participants used antipsychotics in the 24 h prior to the first session (66.7%), balanced between groups (64.7% active vs 69.2% sham). The majority of patients were either alert or drowsy, with 83% having a RASS score of 0 or -1.

**Table 1:** Baseline demographics and clinical characteristics. Variables are mean (SD) unless mentioned otherwise. Note: during the baseline visit, presence of previous delirium (ever) and clinical delirium was based on information reported by the legal representative and electronic patient record review. ASA: American Society of Anesthesiologists; ICDSC: Intensive Care Delirium Screening Checklist; ICU: Intensive Care Unit; RASS: Richmond Agitation and Sedation Scale; UMC: University Medical Center.

	Overall (N = 30)	Active (N = 17)	Sham (N = 13)
*Baseline demographics*			
Center, n (%)			
*HagaZiekenhuis*	12 (40.0%)	7 (41.2%)	5 (38.5%)
*Radboud UMC*	5 (16.7%)	3 (17.6%)	2 (15.4%)
*UMC Utrecht*	13 (43.3%)	7 (41.2%)	6 (46.2%)
Age	74.2 (9.94)	73.1 (10.5)	75.7 (9.33)
Female, n (%)	5 (16.7%)	2 (11.8%)	3 (23.1%)
Body mass index	27.1 (3.89)	27.3 (3.14)	26.9 (4.78)
ICU, n (%)	17 (56.7%)	9 (52.9%)	8 (61.5%)
Postoperative, n (%)	19 (63.3%)	9 (52.9%)	10 (76.9%)
Cardiovascular disease, n (%)	24 (80.0%)	12 (70.6%)	12 (92.3%)
Neurovascular disease, n (%)	8 (26.7%)	3 (17.6%)	5 (38.5%)
Diabetes, n (%)	7 (23.3%)	3 (17.6%)	4 (30.8%)
Previous delirium, n (%)	5 (16.7%)	3 (17.6%)	2 (15.4%)
*Clinical characteristics at randomization*			
Clinical frailty score	3.59 (1.60)	3.93 (1.79)	3.17 (1.27)
ASA classification, n (%)^a^			
*2*	3 (10.0%)	1 (5.9%)	2 (15.4%)
*3*	15 (50.0%)	10 (58.8%)	5 (38.5%)
*4*	7 (23.3%)	4 (23.5%)	3 (23.1%)
*5*	1 (3.3%)	1 (5.9%)	0 (0%)
Estimated duration delirium before randomization, in days, median [IQR]	4.5 [3, 7]	6 [3, 10]	3 [3, 5]
Intubated, n (%)	4 (13.3%)	2 (11.8%)	2 (15.4%)
ICDSC score^b^	5.27 (0.94)	5.35 (0.86)	5.15 (1.07)
Medication in last 24 h, n (%)^c^			
Antipsychotics	20 (66.7%)	11 (64.7%)	9 (69.2%)
Benzodiazepines	5 (16.7%)	3 (17.6%)	2 (15.4%)
Opioids	8 (26.7%)	4 (23.5%)	4 (30.8%)
Other sedatives	8 (26.7%)	5 (29.4%)	3 (23.1%)
RASS score, n (%)^b^			
-*2*	3 (10.0%)	1 (5.9%)	2 (15.4%)
-*1*	13 (43.3%)	9 (52.9%)	4 (30.8%)
*0*	12 (40.0%)	6 (35.3%)	6 (46.2%)
+*1*	2 (6.7%)	1 (5.9%)	1 (7.7%)

a ASA is missing in 4 patients.

b Assessed during baseline visit.

c Medication administered in the 24 h before the first tACS session. Antipsychotics included haloperidol, quetiapine, clozapine; benzodiazepines included midazolam, lorazepam, temazepam, oxazepam; other sedatives included: propofol, esketamine, zopiclone, clonidine.

### Feasibility

**Fig. 3:**
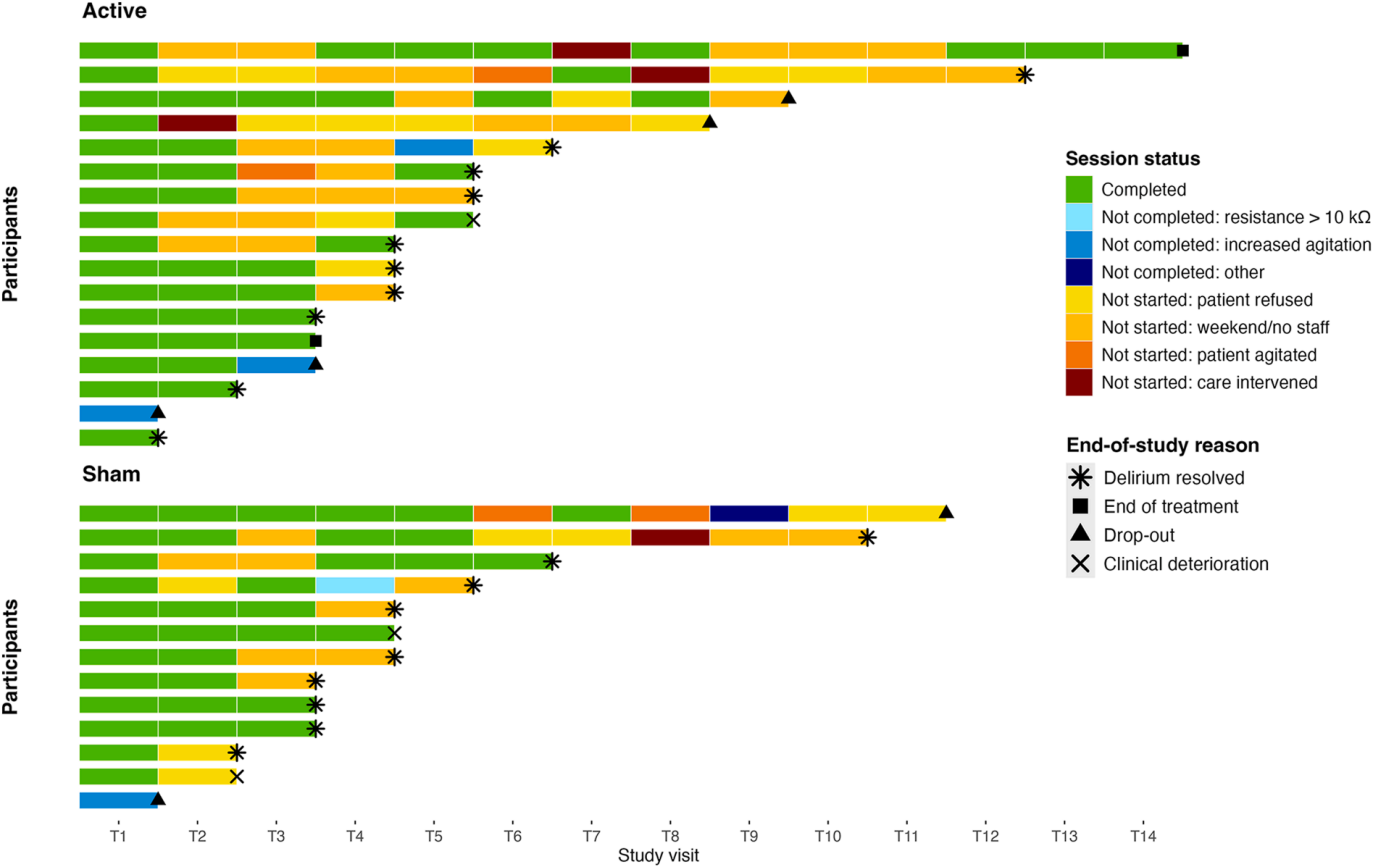
Session status per study visit. Each row represents one participant; each column a scheduled visit (T1–T14). Tile colors indicate the session status at each visit. End-of-study symbols mark the reason for treatment discontinuation. ‘End of treatment’ refers to reaching the maximum 14 days or hospital discharge. Participants are shown separately for the active (top) and sham (bottom) stimulation conditions.

The first session completion rate was 93% (95% CI: 78–99%). When stratified by group, first session completion rate was 94% in the active group (16/17; 95% CI: 71–99%) and 92% in the sham group (12/13; 95% CI: 64–99%). Participants in both groups had a median of three eligible treatment days (IQR: 2–4). The active group completed a median of two tACS sessions (IQR: 2–3, range: 0–8) and the sham group a median of 3 (IQR: 2–4, range: 0–6), corresponding to a treatment adherence rate of 69% and 73%, respectively. Of scheduled sessions, the most common reason for non-delivery was weekend/no-staff availability (24/89 [27%] active; 10/58 [17%] sham), followed by patient refusal (12/89 [14%]; 7/58 [12%]). Non-delivery attributable to agitation or clinical instability was infrequent (active 5/89 [6%]; sham 3/58 [5%]) (Fig. 3, Supplementary Material 2). Individual study endpoints were mostly delirium resolution, namely 59% in the active group and 69% in the sham group (Fig. 3). Two completed tACS sessions in the sham group were paused for 2–3 min to reapply conductive gel to the tACS electrodes due to a too high impedance. Apart from these instances, no completed tACS sessions were paused or modified.

### Safety

One participant died within the intervention period due to multiorgan failure caused by sepsis, which was considered unrelated to the study intervention. No SAEs were attributed to the intervention in either group. The overall incidence of AEs was comparable between groups: 6 of 17 participants (35%) in the active group and 4 of 13 (31%) in the sham group experienced at least one AE. AEs in the active group included several events possibly related to tACS, all occurring during stimulation: removal of a nasogastric tube (n = 1), EEG cap removal 5 min into the stimulation (n = 1), increased agitation at stimulation onset (n = 1), and systemic body heat (n = 1), as well as two occurrences considered unrelated to tACS: transient cardiac arrhythmia in a patient with a known history of rhythm disorders near the end of stimulation (n = 1) and EEG cap removal prior to tACS onset (n = 1). In the sham group, AEs included two occurrences possibly related to study procedures and occurring during stimulation: a request to stop treatment near the end of stimulation due to feeling feverish (n = 1) and panic with shortness of breath (n = 1), and one unrelated occurrence of finger pain reported the following day. Additionally, one participant in the sham group experienced persistently elevated stimulation impedance, resulting in early termination of the session, which was also recorded as an AE. No AE required pharmacological treatment or physical restraint. Management consisted of discontinuing the stimulation, except for the patient who experienced panic, where the treating nurse initiated breathing exercises.

### Tolerability

In the active group, 6% of tACS sessions were not completed once started, compared to 8% in the sham group. No tACS-related sensations met the criteria for AEs as described in section 2.5. Among participants who were able to report subjective sensations, side effects were mild and occurred at broadly similar rates across groups. The most commonly reported sensations were itching (23% active vs. 10% sham) and heat under the electrode (15% active vs. 20% sham). Other sensations, including neck pain, iron taste, burning sensation, and phosphenes, were each reported by no more than one or two participants per group (Table 2). Four participants in the active group (24%) and three in the sham group (23%) were unable to report sensations.

**Table 2:** Primary and secondary outcomes. AE: adverse event; SAE: serious adverse event; tACS: transcranial alternating current stimulation.^a^: percentage based on total number of participants able to report sensations.

Outcome	Active (n = 17)	Sham (n = 13)
**Feasibility**		
First session completion rate, n (%)	16 (94%)	12 (92%)
Eligible treatment days, median [IQR]	3 [2–4]	3 [2–4]
Completed tACS sessions, median [IQR]	2 [2–3]	3 [2–4]
Treatment adherence rate, % (95% CI)	69% (57–79)	73% (59–83)
tACS sessions started but not completed, n (%)	3 (6%)	3 (8%)
**Safety**		
SAE possibly related	0 (0%)	0 (0%)
Non-related SAEs	1 (6%)	0 (0%)
AE possibly related	4 (23%)	2 (15%)
Non-related AEs	2 (12%)	1 (8%)
Device deficiency	0 (0%)	1 (8%)

**Tolerability (tACS sensations)**^a^	**Active (n** = **13)**	**Sham (n** = **10)**

Itching	3 (23%)	1 (10%)
Pain	0 (0%)	0 (0%)
Burning sensation	0 (0%)	2 (20%)
Heat under electrode	2 (15%)	2 (20%)
Iron taste	0 (0%)	1 (10%)
Headache	0 (0%)	0 (0%)
Neck pains	1 (8%)	1 (10%)
Phosphenes	1 (8%)	0 (0%)
Dizziness	0 (0%)	0 (0%)
Unable to report	4 (24%)	3 (23%)

### Blinding

Researchers guessed that sham stimulation was administered in 19 of 30 first tACS sessions, while active stimulation was guessed 11 times. Overall, the researchers correctly identified the treatment allocation in 15 of 30 trials (50%).

## Discussion

This multicenter randomized double-blind sham-controlled pilot study represents, to our knowledge, the first investigation on feasibility, safety and tolerability of tACS as a treatment for patients with delirium. The primary objectives were met, demonstrating that daily administration of tACS in a heterogeneous cohort of hospitalized older adults with delirium is both feasible and safe. The intervention achieved a high first session completion rate of 92–94%. Treatment adherence rate was approximately 70% in both treatment groups. The safety profile was comparable between groups with low (S)AE incidences. Tolerability was favorable, with some participants experiencing only mild tACS-related sensations and 6–8% of tACS sessions not completed once started. The longer estimated delirium duration before randomization in the active group should be considered when interpreting descriptive between-group differences. This pilot study was designed to assess feasibility and safety and therefore, no conclusions can be drawn on whether tACS is able to modify brain activity underlying delirium or contributes to clinical recovery.

Regarding feasibility, the high first session completion rate demonstrates that tACS can be successfully initiated in the majority of delirious patients, even in the context of the additional procedural burden of pre- and post-stimulation EEG recordings. The observed gap between the high first session completion rates and the lower number of repeated treatment sessions is noteworthy. This finding reflects that, although tolerability of tACS sessions was favorable, maintaining repeated daily treatment sessions was challenging due to practical and logistical demands in this clinical setting. In a home treatment RCT of tACS in 255 major depressive disorder (MDD) patients, between 83% and 86% of subjects reported full twice-daily adherence during a 14-day period [37]. However, this comparison is limited by fundamental differences in population and setting, as the MDD cohort comprised ambulatory, cognitively intact patients self-administering treatment at home.

No SAEs were attributed to the stimulation, and the overall incidence of AEs was low. However, the active group showed a numerically higher rate of AEs possibly related to the intervention (23% vs. 15%). It is important to note that the current safety profile reflects a median of 2-3 tACS sessions per participant. If future trials succeed in improving adherence and delivering more tACS sessions, the cumulative exposure and the safety profile may change. Continued systematic safety monitoring will therefore be essential in subsequent studies. The observed safety data are consistent with the most recent international safety guidelines. Antal et al. reported that across over 300,000 tES sessions involving healthy individuals and clinical populations, no SAEs related to stimulation have been documented [38].

Similar to our findings, Antal et al. reported that sensations such as tingling and itching are the most common effects related to treatment with tACS, and that these are generally mild and occur at similar rates in active and sham conditions [38]. A concern related to tACS is the induction of retinal phosphenes, subjective perceptions of flickering light caused by volume conduction to the retina [25]. In healthy cohorts, 10 Hz stimulation is known to lower the threshold for phosphene perception significantly [39]. Only one participant, in the active group, reported phosphenes in this pilot study. In healthy younger adults phosphene reporting can approach 100% under specific montages and lighting conditions [40]. Several factors may account for this discrepancy. The core features of delirium, inattention and altered awareness, may reduce the patient's ability to report subtle sensations such as phosphenes, likely leading to underestimation of this result in our study. This notion is supported by our finding of 23% of patients being unable to report on tACS sensations. Trained research staff did not observe overt behavioral signs of distress or sustained agitation during stimulation. Thus, potential unreported perceptual effects cannot be excluded, but they were not associated with clinically apparent distress during monitored sessions.

Recruitment proved challenging throughout the pilot phase and warrants careful consideration for future trials. Of 167 patients referred for eligibility assessment, only 30 (18%) started treatment. The largest sources of attrition were patient or family refusal and delirium resolving before informed consent or randomization could be completed. These numbers are no exception in delirium trials, such as the EuRIDICE trial [41], as the condition is often transient and may fluctuate over time, meaning that the window for inclusion is often narrow. The slow enrollment pace, approximately 30 patients over 18 months across three centers, underscores that large-scale trials in this population will likely require longer recruitment periods, expanded site participation, or adaptations to the consent procedures, such as simplified surrogate-consent pathways and, where ethically and legally permissible, deferred-consent approaches. Embedding research activities within routine clinical work may further facilitate timely screening and enrollment beyond standard research staffing hours and reduce missed recruitment opportunities.

The primary strength of this study is its rigorous design as a multicenter, randomized, double-blind, sham-controlled trial. The successful implementation of blinded outcome assessments and the objective verification of blinding success distinguish this study from many early non-invasive brain stimulation trials which often lack robust control conditions [42]. However, several limitations must be acknowledged. Almost a quarter of intervention participants were unable to report on tACS sensations. This finding could impact the reliability of our tolerability findings, though trained research staff who stayed with the patient throughout the whole tACS session did not report visual signs of distress. The inclusion of patients across ICU and non-ICU wards, postoperative and non-postoperative settings, and with varying underlying medical etiologies is noteworthy. The heterogeneous sample enhances external validity, demonstrating that tACS can be applied across diverse clinical settings. However, it also limits mechanistic and therapeutic interpretation since the study was not designed or powered to determine whether tACS has differential effects across delirium subtypes, etiologies, illness severity, or delirium duration. Furthermore, with 57% of participants being ICU patients, the sample may not fully represent the broader delirium population. This likely reflects the practical reality that ICU patients are more continuously monitored and thus more easily identified for inclusion. Nonetheless, our results in this severely ill cohort are promising when the high clinical demands are considered.

In conclusion, this pilot study demonstrates that frontal-occipital 10 Hz tACS can be safely initiated and completed in hospitalized older patients with delirium, with a high first session completion rate and no stimulation-related serious adverse events during a short-course tACS exposure. However, delivery of repeated daily tACS sessions was challenging due to patient-related factors and logistical constraints. The current study established that tACS can be administered in this complex patient population and provides a foundation for larger, adequately powered trials to investigate whether tACS has measurable neurophysiological or clinical effects in patients with delirium.

## Statement concerning the use of artificial intelligence

The authors used Claude (Anthropic, version Claude Sonnet 4.6) for proofreading and language editing of this manuscript. All AI-assisted content was reviewed and verified by the authors. The authors take full responsibility for the integrity and accuracy of the published work.

## Credit statement

Conceptualization: A.J.C. Slooter, I. Tendolkar, W. de Haan, D.J.L.G. Schutter, M.H. Emmelot-Vonk.

Methodology: A.J.C. Slooter, D.J.L.G. Schutter, W. De Haan, E. Van Dellen.

Investigation: J. van der A, D.Y. Lodema, T.H. Ottens, A.J.C. Slooter, M. van den Boogaard.

Data Curation: J. van der A, D.Y. Lodema.

Formal Analysis: J. van der A, D.Y. Lodema.

Project Administration: J. van der A, D.Y. Lodema, T.H. Ottens, A.J.C. Slooter, I. Tendolkar.

Supervision: A.J.C. Slooter, T.H. Ottens, E. van Dellen, W. de Haan, I. Tendolkar.

Funding Acquisition: I. Tendolkar, A.J.C. Slooter, W. de Haan.

Writing – Original Draft: J. van der A, D.Y. Lodema.

Writing – Review & Editing: E. van Dellen, I. Tendolkar, M.H. Emmelot-Vonk, D.J.L.G. Schutter, W. de Haan, M. van den Boogaard, T.H. Ottens, A.J.C. Slooter.

## Declaration of competing interest

The authors declare that they have no known competing financial interests or personal relationships that could have appeared to influence the work reported in this paper.
