# Supplementary material for: Transcranial alternating current stimulation as treatment for delirium: A multicenter randomized double-blind sham-controlled pilot study on safety and feasibility
Source: Neurotherapeutics. 2026 Aug 4;23(5):e01037. doi: 10.1016/j.neurot.2026.e01037 (PMC13469962; doi:10.1016/j.neurot.2026.e01037)
Supplement: Supplementary file 1 [file mmc1.docx]

**Supplementary Material 1****Figure 1.** Placement of the frontal-occipital tACS electrodes. Standardization of the frontal-occipital tACS montage was achieved by positioning the stimulation electrodes directly on the scalp underneath a 64-channel electroencephalography (EEG) cap. The frontal electrode was placed at AFz (left panel), while the occipital electrode was positioned at Oz (right panel), according to the international 10-10 placement system. This setup allowed for consistent electrode localization across study days and participants and also enabled the acquisition of pre- and post-tACS EEG data, without having to remove the EEG system.

**Supplementary Material 2**

| **Session status** | **Active, n (%)** | **Sham, n (%)** |
| --- | --- | --- |
| Completed | 45 (51%) | 35 (60%) |
| Not started: weekend / no research staff | 24 (27%) | 10 (17%) |
| Not started: patient refused | 12 (14%) | 7 (12%) |
| Not started: patient agitated | 2 (2%) | 2 (3%) |
| Not started: care intervened | 3 (3%) | 1 (2%) |
| Not completed: increased agitation | 3 (3%) | 1 (2%) |
| Not completed: impedance >10 kΩ | 0 (0%) | 1 (2%) |
| Not completed: other | 0 (0%) | 1 (2%) |

**Supplementary Table 1. Session status across all scheduled treatment sessions, by group.** Each scheduled session corresponds to one participant study-day (T1-T14) and was assigned a single status. Percentages are of all scheduled sessions within each group (active, n = 89; sham, n = 58). Completed: the full 30-minute stimulation was delivered. Not completed: the session was started but stimulation was not finished (impedance >10 kilo-ohm (kΩ), increased agitation, or other). Not started: the session was not initiated (weekend/no research staff, patient refusal, patient agitation, or care intervened).
